# Cytomegalovirus and Aging—How Can the Field Move Forward?

**DOI:** 10.1093/infdis/jiag099

**Published:** 2026-02-28

**Authors:** Michael Boeckh, Lawrence Corey

**Affiliations:** Vaccine and Infectious Disease Division, Fred Hutchinson Cancer Center, Seattle, Washington, USA; Department of Medicine, University of Washington, Seattle, Washington, USA; Vaccine and Infectious Disease Division, Fred Hutchinson Cancer Center, Seattle, Washington, USA; Department of Medicine, University of Washington, Seattle, Washington, USA

**Keywords:** CMV, biological aging, EBV, immunosenescence, herpesviruses, neurocognitive decline, pathogen persistence

**(See the Major Article by Momkus et al on pages e1320–9.)**

The impact of cytomegalovirus (CMV) on the human immune system and clinical outcomes associated with aging has fascinated researchers since the late 1990s. There has been increasing evidence that CMV has a profound impact on the immune system, accelerates immunosenescence and upregulates aging biomarkers [1]. Multiple studies have reported an association between CMV seropositivity or higher binding antibody levels with adverse clinical outcomes involving cardiovascular disease, neurocognitive decline, dementia, and frailty [2–4]. However, findings have been conflicting for some end points, such as cardiovascular and overall mortality [5]. Herpesviruses, and CMV in particular, have also been associated with diminished vaccine responses in older adults. This effect is, however, not universal across all vaccines and studies [6–8]. Adding to the complexity, CMV seropositivity has also been associated with beneficial vaccination outcomes for influenza in middle-aged adults and with lower disease incidence in some immunologically related diseases, such as multiple sclerosis [9, 10]. While CMV is a key pathogen in the aging literature, exposure to other herpesviruses (ie, herpes simplex virus [HSV], Epstein-Barr virus [EBV], and human herpes virus 6 [HHV-6]) and potentially other pathogens, either alone or cumulatively, may also increase the risk of cardiovascular disease, neurocognitive or functional decline, and mortality [11, 12].

In this issue of *The Journal of Infectious Diseases*, Momkus et al set out to define associations between chronic pathogens (including CMV, HSV-1, EBV, and *Helicobacter pylori*) with biological aging markers and immunosenescence [13]. Using the National Longitudinal Study of Adolescent to Adult Health (Add Health) US cohort, the authors gathered interviews, physical examinations, and dried blood spot collection from 2 waves (wave IV, median age 28 years and wave V, median age 38 years) to create their study population of 935 persons. The ages 28–38 years represent an age range of persons not typically assessed for chronic pathogen age-related associations. Biological aging was measured by CpG methylation to estimate second-generation epigenetic clocks and immunosenescence by calculating CD4 and CD8 T-cell ratios and of memory to naive ratios of CD4 and CD8 T cells. The authors found that CMV seropositivity was associated with higher epigenetic age acceleration and higher CD4 and CD8 memory to naive ratios, as well as lower CD4 to CD8 ratios, indicative of immunosenescence and biological aging [13]. CMV showed the most consistent relationship with epigenetic age acceleration, with HSV-1, EBV, and *H. pylori* exhibiting lesser associations. An unexpected finding was that *H. pylori* immunoglobulin G (IgG) levels were associated with a higher CD4 to CD8 ratio, suggestive of a younger immune profile. This study is among the first to evaluate markers of aging associated with persistent infections in a premidlife cohort, highlighting the potential impact of persistent infections on early onset biological aging prior to the inception of age-related diseases.

After more than 25 years of largely observational and correlative research, we believe it is time to define a path forward to obtain the necessary data to design definitive clinical trials about the association between CMV disease and premature aging, immunosenescence, and/or neurodegenerative disease. There have been very encouraging recent reports towards this goal. Based on murine studies demonstrating that prolonged high-dose acyclovir can reverse CMV-associated immunosenescence phenotype, investigators in the United Kingdom showed in a randomized pilot trial that 6 months of high-dose valacyclovir reduced viral shedding and led to improved immunogenicity of pneumococcal vaccination [14, 15]. Analyses of large datasets showed associations between varicella zoster virus vaccination and antiviral drugs used for other clinical indications reduced risk of neurocognitive decline and dementia [16, 17]. However, one randomized trial failed to demonstrate improvement of early symptomatic Alzheimer disease in HSV-seropositive individuals using valacyclovir [18]. Perhaps most intriguingly, a recently presented randomized trial of a 48 week course of the antiviral CMV drug letermovir among CMV-seropositive people with human immunodeficiency virus type 1 (HIV) showed a signal of improved inflammatory markers and frailty indices [19]. Finally, mathematical modeling of even partially effective CMV interventions suggests a positive impact on slowing the development of aging-related diseases [20].

Despite this progress, important research gaps continue to exist. While there is compelling evidence that chronic inflammation is a key driver of clinical consequences of aging, the mechanistic link to pathogen active replication or pathogen persistence is poorly established. Current evidence that CMV reactivation rates increase with age come from cross-sectional studies and are often based on indirect evidence from increased antibody levels [21, 22]. The viral load kinetics, risk factors, and mechanistic pathways of viral reactivation measured longitudinally by quantitative polymerase chain reaction (PCR), including a potentially bidirectional relationship with inflammation and their relationship to age, comorbidities, immunosuppression, other pathogens, and vaccine responses, continue to be a black box. Detailed analyses of pathogen reactivation kinetics and the benefit of the reduction in viral reactivation with long-term antivirals have played a pivotal role in the development of therapeutic strategies for direct effects of CMV and HSV in both immunocompetent and immunocompromised individuals [23, 24]. Similar studies are needed for truly defining the link to aging in both the general and immunocompromised populations. Additionally, research is needed to determine whether current antiviral modalities are sufficient to reduce inflammatory and aging-related biomarkers.

We acknowledge the complexities and risk in such studies. Whether single or multiple pathogens should be targeted is uncertain, and more detailed estimates are needed of the attributable risk increase associated with specific pathogens for complex clinical aging phenotypes with multiple progression pathways [25, 26].

Antiviral suppression trials to improve immunogenicity of vaccination in the elderly could be performed with letermovir, which is well tolerated and more potent against CMV than valacyclovir. Clinical end points are ultimately the real proof of efficacy. Neurocognitive decline and frailty are clinically important primary end points [27, 28]. Neurocognitive decline has been used as a primary end point in several recent trials in the setting of early Alzheimer disease or dementia [18, 29]. Because the effect of prior pathogen exposure is likely only contributory to the overall decline associated with aging and recent trials modifying the trajectory of the neurocognitive decline in clinically manifest early disease were modest or unsuccessful [18, 29], any possible intervention directed at pathogens to slow the pace is likely most effective at the preclinical stage in individuals at increased risk, an approach requiring a larger study population and longer duration of therapy, follow-up, and subsequent cost.

With the global rise in life expectancy, the number of aging people experiencing frailty, neurocognitive decline, and dementia is already a pressing health policy problem, which is forecasted to accelerate dramatically over the next 25 years unless modifiable risk factors are proactively addressed [30]. The acceleration of interventional trials to raise pathogen-related research in aging to the next level will require a significant investment but the return to society could be transformative as it may establish targeted antimicrobial interventions as a novel principle of a comprehensive healthy aging strategy, and may also inject energy and investment in the development of novel single- or multitarget antimicrobial agents.
